# Identification of Molecular Targets and Anti-Cancer Agents in GBM: New Perspectives for Cancer Therapy

**DOI:** 10.3390/brainsci13071078

**Published:** 2023-07-17

**Authors:** Agata Grazia D’Amico, Celeste Caruso Bavisotto, Assunta Virtuoso

**Affiliations:** 1 Department of Drug and Health Sciences, University of Catania, 95123 Catania, Italy; agata.damico@unict.it; 2 Department of Biomedicine, Neurosciences and Advanced Diagnostic (BiND), Human Anatomy Section, University of Palermo, 90127 Palermo, Italy; 3 Laboratory of Neuronal Networks Morphology and System Biology, Department of Mental and Physical Health and Preventive Medicine, University of Campania ‘‘Luigi Vanvitelli”, 80138 Naples, Italy; assunta.virtuoso@unicampania.it

The authors of the present literature piece were invited to participate in the present Special Issue at the beginning of 2022, and we were all very enthusiastic at the prospect of assembling a series of articles on new molecular targets and anti-cancer agents in glioblastoma multiforme (GBM).

In the Special Issue entitled “*The Identification of molecular targets and anti-cancer agents in GBM: new perspectives for cancer therapy*” published by MDPI, we have edited a collection of five papers including three original articles and two communications that provide new insights in the following themes: (i) the identification of new molecular targeted and/or prognostic biomarkers for GBM; (ii) recent advances in combined therapy compared to existing gold standard treatment; and (iii) ongoing clinical trials for the treatment of GBM.

Glioblastoma multiforme (GBM), also known as grade IV astrocytoma, represents the deadliest form among brain tumors affecting adult patients and is characterized by a poor prognosis. The existing therapeutic approach consists of a combination of surgery, radiotherapy, and chemotherapy. The pharmacological treatment includes temozolomide which unfortunately has several side effects such as the myelosuppression. To date, efficacious combined therapies as well as the identification of non-invasive alternatives to standard diagnostic approaches are lacking.

The first communication by Della Pepa et al. [1] contributes in advancing the surgical approaches. The authors highlighted the operative nuances regarding the use of 5-ALA in fluorescence-guided tumor resection focalized for teaching purposes. The authors present an interesting overview regarding their routine based on 5-ALA-guided procedures and provide five surgical techniques to ease the surgical workflow: 1. the analysis of visualization, overall workflow, and technical recommendations to improve intraoperative set-up; 2. techniques to reduce the risk of inadvertent residuals and failure to evocate fluorescence; 3. the analysis of specific surgical conditions favoring remnants; 4. the assessment of different degrees of fluorescence and their surgical meaning; and 5. the analysis of false positive cases.

This study represents a very important contribution to the field, reporting on many years of experience of technical strategies that could be useful to increase the use of 5-ALA in patients undergoing surgery for high-grade gliomas. Due to the limitations and difficulties of the surgical approach for high-grade gliomas (HGGs), the employment of 5-ALA provides real-time tumor visualization, and it has become an indispensable surgical technique and standard of care at many neurosurgical departments around the world. However, it is not a technique without limitations. Therefore, reports relating to direct surgical experience are interesting and useful for stimulating research aimed at improving the surgical approach for GBM.

The contribution by Evers et al. [2] deals with changes in the miRNA expression profiles in patient-derived glioblastoma stem-like cells (GSCs) and their differentiated status as adherent GBM cells. By using a polymerase chain reaction array, the authors identified 31 dysregulated miRNAs. About 10 highly regulated miRNAs, including miR-425-5p, miR-17-5p, miR-223-3p, and the let-7 miRNA family, could be considered as promising miRNA candidates to manipulate the differentiation status of glioblastoma stem-like cells (GSCs). Regarding the heterogeneity of GBM, data in the literature are varied, and various interpretations have been proposed. Among them, GSCs are considered principal modulators of the tumor microenvironment as well as the origin of radioresistance and chemoresistance. The role of GSCs in tumor microenvironment manipulation has been largely studied, and a unique miRNA expression pattern has been observed. As stated by the authors, miRNAs can mediate many critical pathways that contribute to cancer progression, and it is of interest that this study investigated changes in the miRNA expression profile in patient-derived sphere-forming GSCs.

This is clinically important since those miRNAs have recently been considered as novel diagnostic and prognostic biomarkers and could represent the main factors able to affect multiple target genes involved in the pathological processes characterizing GBM.

The question of oncogenic pathways’ involvement in GBM was addressed in a study conducted by Gallo et al. [3], which focused on a protein encoded by the gene YWHAB, 14-3-3β, which is commonly upregulated throughout the initiation and progression of GBM. Malignant proliferation is an important characteristic of cancer cells; therefore, the 14-3-3β family’s regulatory effects on tumor cell proliferation have been largely investigated. The authors demonstrated how the inducible knockout of 14-3-3β reduced the proliferation and spheroid formation in a human GBM cell line, U87MG. The authors strongly suggested the use of 14-3-3β knockout to investigate its role in the initiation and progression of GBM. Data are further supported by a spheroid 3D model of U87MG cells, which is a more representative system of the in vivo tumor.

Differential diagnosis issue was raised in a study by Guo et al. [4], who focused on a new approach based on single-cell resolution to reveal the heterogeneity of cancers. Indeed, the heterogeneity of cancer has a profound impact on the prognosis of patients. The authors used scRNA-seq analysis to explore the diversity and similarities in the occurrence and development of pediatric- and adult-type GBM. The number of clusters in the adult group was significantly increased as compared to the pediatric group, revealing that adult-type GBM possesses higher heterogeneity than the pediatric one. These results shed light on one of the main applications of scRNA-seq, which is the study of the differentiation and development of tumor cells. Also important is the possibility to investigate some of the key molecules during the acquisition of malignant potential for GBM cells. It is noteworthy that the authors identified ASTN2 as a migration biomarker in adult GBM. ASTN2 encodes for astrotactin, and it was shown to increase the migration ability of GBM cells, shedding light on a new potential therapeutic target.

The study of genetic and molecular profiles of single cells is the subject of another article included in this Special Issue—the study by Thakur et al. [5], which found an interesting link between endothelial dysfunction in COVID-19 infection and its impact on GBM progression. The authors analyzed the expression profiles of key players in innate immunity and inflammation between brain endothelial dysfunction caused by COVID-19 and GBM progression by using a single-cell transcriptome obtained from the gene expression omnibus (GEO). Data analysis revealed a substantial overlap between COVID-19 and GBM in the context of endothelial dysfunction. The authors highlighted that endothelial dysfunction caused by COVID-19 permits the virus to cross into the brain, causing inflammatory events that increase the risk of GBM.

The studies discussed herein highlight crucial insights into the molecular mechanisms, treatment modalities, and prognostic factors associated with GBM malignancy. The clinicians’ contributions provide a very important tool to improve the current technical strategies. Moreover, the evidence from the other manuscripts identify new molecular targets as well as new correlations between possible infection or dysregulated gene expression and GBM outcomes. In this regard, the studies included in the present Special Issue strongly suggest the use of miRNAs as candidates to manipulate the differentiation status of GSCs, which are well known for being hard to target. These cells can escape the gold-standard therapy since they possess innate clonogenic differences. 

In conclusion, this Special Issue entitled “The Identification of molecular targets and anti-cancer agents in GBM: new perspectives for cancer therapy” sheds light on the advancements in GBM research.
